# A Multidimensional View of Personal Health Systems for Underserved Populations

**DOI:** 10.2196/jmir.1355

**Published:** 2010-08-04

**Authors:** Thomas A Horan, Nathan E Botts, Richard J Burkhard

**Affiliations:** ^2^Department of Management Information SystemsCollege of BusinessSan Jose State UniversitySan JoseUSA; ^1^Kay Center for E-Health ResearchSchool of Information Systems and TechnologyClaremont Graduate UniversityClaremontUSA

**Keywords:** Medically underserved, chronically ill, personal health records

## Abstract

The advent of electronic personal health records (PHR) provides a major opportunity to encourage positive health management practices, such as chronic disease management. Yet, to date there has been little attention toward the use of PHRs where advanced health information services are perhaps most needed, namely, in underserved communities. Drawing upon research conducted with safety net providers and patients, the authors propose a multi-level analytical framework for guiding actions aimed at fostering PHR adoption and utilization. The authors first outline distinctive user and technical requirements that need to be considered. Next, they assess organizational requirements necessary to implement PHRs within health systems bound by limited resources. Finally, the authors analyze the overriding health care policy context that can facilitate or thwart such efforts. The conclusion notes that heightened national attention toward health information technology and reform provides a significant opportunity for initiatives whose goal is to increase widepread access to PHRs.

## Introduction

### The Need to Extend Public Health Record System Usage to Underserved Populations

There are important and pressing reasons for providing personal health information to underserved populations. Underserved groups are widespread within the United States and other industrialized countries [1,2] resulting in broad inequities in many communities. Such populations are typically diverse, low-income groups who lack adequate access to traditional care and are often referred to as living in the "safety net" [3]. Due to the fragmented nature of this population’s health care background and a general lack of preventive health measures, individuals in the safety net (including low income, uninsured, homeless, and other special needs groups [4]) are taxing general health and emergency health systems to their limits, resulting in adverse fiscal impacts on providers as well as private and governmental payers [5]. Outside of increasing medical staff and resources, one of the most promising ways to help alleviate the stress incurred by ever evolving patient needs and the health care systems that support them is to increase people’s ability to manage their own health.

Over the last few years, electronic and Web-based personal health record systems (PHRs) have entered the marketplace and have begun to demonstrate value for health care consumers. PHRs allow the patient to manage personal health information, to maintain provider and insurance services, to manage prescriptions, appointments, and medical procedures, and to receive data from the electronic medical record (EMR) systems used by providers to manage and process medical services provided to patients [6-9]. In addition, PHRs can help patients maintain a continuous health record for themselves and family members and also to communicate more extensively with physicians and other health care providers [9]. PHRs and the related health information technologies provide a new level of patient health management with the opportunity for an available, cumulative health management record. The success of large PHR implementations by Kaiser Permanente (My Health Manager) and the Veterans Health Administration (My Health*e*Vet), coupled with the recent entry of Web solution firms such as Microsoft and Google, leave little question as to the growing influence that PHRs will have on the future of health care transactions [8]. Our perspective is that the underserved should not be left out of these exciting advances in health technology, and conversely, there is value in extending the reach of PHRs in this direction.

While the emergence of PHRs provides an opportunity to address numerous challenges related to the personal management of health, this can only occur if such systems are usable by and accessible to a diverse array of health care consumers. To this end, a research-based, analytical framework has been designed that provides actionable guidelines for developing PHRs for the underserved. The framework described here focuses on the unique challenges of providing practical PHRs for underserved groups and outlines concurrent actions that need to be taken at the technology, provider, and policy levels. Prefacing this framework is an overview of the unique need for PHRs within the underserved groups, the research that has been conducted within these settings, and the overall findings that have informed what is being forwarded as a “framework for action” toward the timely design and adoption of PHRs within vulnerable populations.

### Enhancing Health Self-Management: A High-Value Target

The US health care system struggles with an increasing burden brought about by an aging population, increasing numbers of underinsured populations, the high incidence of chronic health conditions, and an insufficient pool of incoming health care workers [10,11]. As a result, the burden of managing patient health, therapy, and medical transactions is shifting from overtaxed providers to patients and their families. In this environment, health management technologies such as PHRs can play a key role in aiding those requiring the most assistance.

Information technologies have gradually transformed many aspects of the health care service enterprise, yet scant attention has been paid to their use within provider settings that focus on diverse and vulnerable populations [3]. Much of the research on PHRs focuses on relatively educated health care consumers such as commercial health plan subscribers who are computer literate and have easy access to the Internet. Some of the most important health care system impacts, however, could be provided by efficient and effective solutions for implementing PHRs within populations not served by existing health care systems [9,12,13]. While it is clear that electronic record keeping and practices can decrease unnecessary medical encounters [14], such efficiencies can also be vital to resource-strapped providers who often care for the most vulnerable populations. Recently, there has been some attention to the need and potential for PHR adoption within one segment of the population: persons with disabilities [15]. A very similar but perhaps broader need for examination exists in the underserved population as well.

The term “underserved” refers to broad and diverse groups who are typically of low socioeconomic status. They are often uninsured or underinsured and are at risk of critical health problems due to gaps in health maintenance. The two most common measures of underserved are those without health insurance and those living in communities deemed by the federal government as medically underserved areas (MUAs) [16]. Recent estimates report that there are some 47 million uninsured Americans in the country, with a greater number of Americans (some 66 million living) in MUAs. In the state of California (where the bulk of this study’s field research was conducted), some 8.5 million Californians currently do not have health insurance, most of whom live in medically underserved areas [16-18]. The recently passed national health care reform legislation will make strides to address this problem, but it will take several years to unfold and does not erase the trying economic and health circumstances for many who have been underserved.

While the research presented here has focused on underserved groups in the United States, similar trends have been identified in many industrialized countries. In several European nations, for example, eHealth services are increasingly embraced among Internet users, while underserved groups in many parts of Europe have lower levels of such access [19-21]. For many, “only well-placed users (high education, high socioeconomic status)” are able to take advantage of eHealth services [22]. It is clear that developing nations in Asia, Africa, Middle East and elsewhere face even more daunting obstacles in providing such access.

Within the United States, many see a pressing need for practical solutions that can enable underserved populations to manage their own health care. In California, approximately 90% of adults who are uninsured do not qualify for public health care assistance; the largest proportion (32%) of this population is of Latino ethnicity [23]. Such individuals often engage in migratory work, lack stable residency, and are unlikely or simply unable to seek consistent preventive health services necessary to identify and address early indicators of health problems [24]. Moreover, fragmented care for chronic conditions such as diabetes can lead to life threatening situations that not only impact the individual but also place an extraordinary burden on the limited health care resources of both the individual and the health care system. Since these patients often lack an identifiable or familiar location to receive health care, there is little opportunity to create relationships with providers, thus decreasing the number of opportunities to receive preventive care [5]. In these situations, the emergency receiving department often becomes the primary care provider due to a lack of known choices, resulting in an expensive and excessive burden on hospital systems. Thus, convenient access to health information resources, appropriately adapted to the underserved patient, can have a positive impact on health and health resources [25]. Accordingly, provision of PHR resources focused on the needs of vulnerable populations deserves greater attention and is what the following research endeavors to accomplish.

As Senator Harkin said in his introduction to a 2009 congressional hearing on health care reform, “prevention and public health have been the missing pieces in the national conversation about health care reform. It’s time to make them the centerpiece of that conversation. Not an asterisk. Not a footnote. But the centerpiece of health care reform. And we need to guarantee that our most vulnerable high-risk populations have equal access to prevention services and public health [26].” The concepts below are offered as a means for PHRs to play a key role in an evolving health information technology (health IT) system.

### A Multi-Phased Research Approach

This research aimed to outline several important ways in which PHRs can assist underserved populations. Also examined were several important obstacles inherent in the adoption of PHR in these communities. Between 2007 and 2008, with support from the Blue Shield Foundation, a series of structured and unstructured expert interviews were conducted. The first phase of interviews (n = 17) consisted of open-ended questions regarding general perceptions of the utility and feasibility of PHRs and included patients, outreach workers, care managers, and medical practitioners who function within the safety net. The second phase of interviews (n = 8) consisted of extensive structured interviews conducted with experienced leaders functioning within systems and policy-related levels [27].

This two-phased approach is consistent with grounded theory methodologies [28,29] and provided the opportunity to explore not only the hard realities of adopting personal health information systems within the safety net environment, but also the opportunity to devise systems that could assist in achieving health care goals. Participants were asked about their general perceptions and recommendations regarding the use of PHRs within the safety net and the inherent barriers and opportunities their use would entail. In addition, field visits were conducted within two underserved settings in California, one rural migrant field-worker environment in Sonoma, California, and one urban underserved setting in Los Angeles. The transcribed text of the interviews was categorically coded and iteratively categorized for thematic analysis by the researchers. The principal thematic outcome of this analysis (described in detail below) was the identification of personal, technical, organizational, and policy related dimensions for consideration when introducing PHRs into underserved settings. Findings and directions were then outlined within each of these dimensions.

As outlined below, key challenges identified through this and related research included user and technically centered challenges in accessing and using technologies, organizational challenges in the adoption and implementation of EMRs and PHRs, and a general lack of governmental policies and associated funding to provide the support for user and organizational adoption. The challenges, issues, and guidelines presented here were discussed and validated in a variety of settings, including PHR conferences and professional meetings [30,31]. Given the current spirit of enacting health care reform, the following elements and framework are presented as considerations for extending personal health management as part of broader health systems and health IT change.

## Findings

The findings from this research have been organized into a deployment framework that synthesizes key issues identified across personal, technology, organizational, and policy related dimensions (Table 1). Based on findings from interviews, field visits, and literature review, each of these dimensions is advanced as requiring attention when considering and implementing PHRs in underserved populations. The following sections outline how these dimensions were defined and identified as central aspects for adoption and implementation of PHRs within underserved communities.

### Challenges for Users: Usability Concerns

The PHR usability and functionality findings identified in this review suggest that some members of underserved populations are aware of tools such as PHRs, but efforts to encourage adoption often fall short due to the inability to engage patients in direct health management behaviors and enable transparent, patient-driven communications between patients and their care providers [32]. Health information is frequently presented in a manner that requires a higher literacy level than many other forms of information [33]; this presents obstacles for underserved subgroups, particularly ethnic minorities and undocumented workers who often lack sufficient formal education to become successful consumers of health information. Literacy shortfalls impact the ability to understand consent forms, to understand clinical instructions, to follow prescriptions, and to manage appointments [34,35]. In addition, this barrier is compounded when conveyed through an information system that people are not experienced in using.

Conversely, there is a motivation among safety net patients to get some control over their health information. For example, in field visits to community clinics, it was identified that many members of underserved groups go to great lengths to keep track of small but important health information items and feel empowered by information resources such as ID cards issued by health providers because they know that having a document or card will increase their credibility with health care providers in future encounters [31].

In terms of overcoming these challenges, designers need to incorporate insights and findings related to human-computer interaction in underserved settings with best practices in health care to create a system that is secure, consumer-centric, and accessible [36]. While the ability of the underserved to access and use health information technology currently represents a barrier, the ability of these same populations to use a cell phone or an automated teller machine (ATM) is widely accepted, even though these tools are representative of complex information technologies. Accordingly, PHRs can be fashioned in such a way as to create equal access and understanding for all vulnerable populations. Designing systems that meet the needs of the most vulnerable users will ensure a wider adoption of health technology tools by the entire population [37].

In addition, there is value in understanding the communities in which the 66 million underserved Americans live and work. For example, interviewees noted that caretaker roles among migrant workers are often occupied by women, who may serve as health managers for multiple generations in a family group. Some families adapt by having a single member of the family obtain health insurance. This family member in turn serves as a channel of information and may even act as a conduit for services and medications for other members of the family. New PHR solutions should be adapted to work within these cultural practices. Moreover, there is a pressing need to connect personal health solutions with the community to address emerging public health issues. For example, asthma is on the rise in underserved communities, and there are currently inadequate means to engage families and thus facilitate the use of preventive measures [38]. There is a significant opportunity to address the complex epidemiology of health conditions in underserved communities through PHRs that address such specific needs.

### Barriers to Access: Getting to the Technology

The value of having a primary medical home (a primary health provider or other resource who retains an individual’s health information and serves as a coordinator for services) is now widely accepted [39]. For underserved populations, it is equally, if not more, important that patients have a “virtual medical home” due to the highly fragmented set of services in this population. However, a distinct obstacle that underserved communities will endure in trying to adopt PHRs is the lack of access to a computer either at home or at work and the related technical experience that comes with ownership. The well-documented “digital divide” still separates underserved communities from information technologies by a technology gap that results from low income, little or no education, misunderstanding of the value or purpose of information technologies, as well as many other limiting factors [40-43]. These conditions, coupled with the challenges of usability, literacy [44], and consistent health care, limit the potential adoption and use of PHRs [9,45,46].

It has been argued that technological diffusion will change this circumstance [47]. However, while overall computer ownership and use have increased at all levels of society, the elderly and populations of the lowest income levels still lag behind despite dramatic decreases in the cost of computer technologies [48,49]. Assuming that access to PHRs is not restricted by cost, other barriers must be considered. Compaine [50] suggested that a key factor in determining adoption of a technology is whether the skill level required to use a particular tool crosses a threshold where it is easy to use by the general population. Similarly, Salvador and Sherry [51] recommended creating technologies that include cultural adaptations for specific populations, especially when considering culturally diverse “low-tech” settings, both domestically and internationally.

The interviews and field visits conducted with safety net providers confirmed these findings and revealed several new issues and opportunities. For example, many experts and target community members emphasized that a prerequisite to access and PHR use was the development of trust, both in the privacy and usefulness of the technology, as well as in the health care system itself. While secure methods for accessing our health records is a widespread concern, such an issue is exacerbated for those who are reliant on accessing information from public resources. Others pointed to opportunities for access by designing systems that allowed for simple health information transactions and the ability to review information over more readily adopted mobile devices such as cell phones. This is of great significance for safety net populations, as they are often highly transient and in need of flexible and more secure methods for managing health information.

### Encountering the Organization: Fragmented Systems

Attempts to implement PHR solutions for the underserved are challenged by a fragmented health care system in which there is limited communication between hospitals, clinics, practitioners, and community-oriented providers. Fragmentation leads to higher costs from duplicated services, as well as the potential health risk that arises from unnecessary medical procedures. In order for PHR systems to be effective for the underserved populations, broad-based participation and collaboration will be needed from all stakeholder and service provider groups. Consistent with this approach are projects such as the MiVIA program in Northern California, which is a PHR system for migrant farm worker populations that has diligently included consumers, health care leaders, and staff of community-oriented organizations in the development of the application with the aim of increasing communication opportunities across all aspects of the service chain [27].

Another challenge for PHR deployment within all populations, including underserved populations, is the high cost of implementing EMRs used by service providers. PHRs will be most valuable to patients if they are highly integrated with EMR systems, but not all health care providers use EMR systems as of yet, and the adoption of such systems has been slow. Despite the recent national push through the American Reinvestment and Recovery Act (ARRA) of 2009 to integrate EMRs into health service organizations, sufficient funding is often lacking for community health providers. The cost for the rollout of an EMR system per clinician can reach tens of thousands of dollars, an amount that does not include the costs of lost productivity as practitioners and health care personnel learn to operate and assimilate new systems into their practice [52]. Recent research findings by the Agency for Healthcare Research and Quality (AHRQ) support this need for understanding the use and value of a system, suggesting that the most effective PHRs will be integrated throughout a patient’s care plan in a manner that informs patients of the health benefits that they will potentially experience through use of the PHR [10]. This, however, cannot happen until the community health providers that support the underserved are able to effectively adopt EMR systems for themselves.

Interviews and site visits to community clinics and safety net providers revealed that there is a reluctance to step forward in providing PHRs for their clientele. The principal reason is financial—these organizations are very hard-pressed for basic EMR resources. There is also reluctance given the diverse population that frequents the clinics. However, active involvement with health care is of great importance for safety net populations and suggests a pressing need to find innovative means for electronically delivering this information in ways that recognize the organizational limitations of the safety net setting. For example, as an outcome of this research, the researchers are actively field testing a PHR designed along the lines of an ATM [53]. The advantage of ATMs is that they are broadly accepted and widely used models of public information systems. There are undoubtedly countless other approaches that could be considered.

### A Way Forward: Policies for Empowering Vulnerable Patients

Given the historic health reform changes presently underway in the US, the time is ripe to advance policies that assist in the implementation of a PHR model for underserved communities. The recently passed health reform legislation has placed near-universal coverage high on the policy agenda and, before that, the passage of ARRA unleashed a wave of national efforts to encourage the broad exchange of health information across local, regional and national healthcare entities. Efforts to increase health information sharing included opportunities for EMR adoption reimbursement, support for Regional (and Local) Extension Centers designed to aid providers in adoption and use of health IT and a host of Beacon Communities to be featured around the country that are composed of providers and their patients who can be models of effective use of health IT [54]. Bolstered by this wave, national and state-level entities, in collaboration with community-based providers, can take active steps to insure that provision of health IT is equally extended to those that serve and are a part of the health safety net.

A cornerstone for these activities is the concept of “meaningful use,” which provides an operational definition for the range of functionalities EMR systems must demonstrate in order to receive federal reimbursement from ARRA [54]. On July 13, 2010, the Centers for Medicaid and Medicare Services released its requirements for such meaningful use by 2011, with some attention to the role of PHRs (mainly as a conduit for providing patient office visit summaries) [55]. Looking past 2011, the Health and Human Services’ Federal Advisory Committee has made recommendations for enhanced patient engagement through meaningful use of PHRs [55]. While the thrust of the requirements and recommendations are incremental, the general direction is to encourage providers to increasingly engage patients through such means. Less clear is how such recommendations will play out within the underserved arena.

The point that is being advanced here is that there is both a clinical and societal rationale for ensuring that underserved populations have ready access to PHRs. From the clinical perspective, PHRs can lead to active engagement in health affairs for a segment of the population that has high rates of chronic disease. From a societal perspective, PHRs may aid in the public health goal of ensuring improved health and health conditions throughout the country. Indeed, the emerging domain of public health informatics has outlined several of the gains that can be made in terms of immunization registries, bio surveillance, and related public health monitoring [56]. These tools will be made that much more valuable by ensuring that the underserved are active participants in these new personal health technologies. That is, the widespread adoption of PHRs could provide an important and missing link toward connecting a population-level focus of public health to the individual circumstance of persons who could benefit from user-focused systems that help manage and potentially prevent chronic conditions. The current push for this in the United States, related to the ARRA of 2009, is in the right direction, but facilitating policy action needs to occur at the local level to weave together integrated service delivery systems and tools.

Interviews and field visits confirmed that the issue of privacy (and trust) is crucial to PHR utilization and is an issue that deserves much attention due to a potential lack of understanding within this population. In particular, there is limited understanding within underserved groups of the degree to which health information can be data mined and the consequences it entails. For example, individuals concerned about issues of residency status or family contact information may be particularly sensitive to information privacy and security due to fears (whether valid or not) of persecution or deportation. While interviewees from these populations had underlying concerns related to their status as noncitizens, the majority of them expressed minimal concerns about providing health information online. (It is perhaps debatable as to whether they truly had minimal concerns or if they did not fully understand the implications of entering health information online). In any case, as new provisions are enacted it will be important to analyze the specific implications for the underserved and how to best ensure that their rights are communicated and upheld. For example, ARRA of 2009 contains provisions that extend and strengthen the Health Insurance Portability and Accountability Act (HIPAA), including implications for PHR vendors [54]. Finding and ensuring that PHR resources targeting the underserved contain both privacy safeguards and affordable means of deployment that enhance trust and privacy will require significant consideration on the part of the community health organizations who seek to adopt these systems.

## Directions

### Framework for Action: PHRs in Underserved Communities

As outlined in the findings above, this research identified four critical layers to consider when devising PHRs for use in underserved populations: personal, technical, organizational, and policy-related dimensions [31]. Within this framework (Table 1) are examples of tangible health IT issues and requirements documented through recent research findings. The framework’s focus on underserved communities distinguishes it from broader, general agendas for electronic health records that have been previously proposed [57]. These findings should guide policy aimed at improving electronic access to personal health information by underserved communities and help to develop appropriate health IT standards and regulations.

**Table 1:** Framework for PHR systems in underserved populations

Conceptual Level	Constructs	Guidelines Relating to the Underserved
Personal	Health management	PHR systems in underserved communities need to address integrated care challenges and bolster continuity of care with proper assessment and maintenance of health outcomes. PHR systems in underserved communities need to include patient education and encouragement toward services needed to engender preventive health maintenance behaviors.
	Language and literacy	PHR systems in underserved communities need to feature multi-lingual capabilities. PHR systems in underserved communities need to be explicitly attuned to limited levels of literacy, computer skills, and health information knowledge.
	Privacy	Privacy and security features need to not only address HIPAA requirements, but also allay concerns unique to underserved populations and provide education on its importance.
Technical	Infrastructure	Low-cost standardized means for effectively importing and exporting patient data across community clinic environments are needed to allow for low-cost architectural approaches. Underlying the adoption of software systems, there is a need for basic technological infrastructure improvements in community provider settings.
	User network	Computer experience and access is limited for the underserved, and, therefore, very user-friendly and publicly accessible interfaces need to be provided. User access requires a range of modalities depending on the type of fixed and mobile access needs and requirements that occur at both the provider and user level. Critical user locations such as emergency rooms require appropriately adapted and efficient interfaces.
Organizational	Administration	As a majority of community health patient data is still paper-based, providers will need incentives to adopt new technologies. Community health organizations need to introduce new workflow and patient communication practices to facilitate PHR use as a health self-management tool.
	Adoption and integration	Community health organizations need increased financial support in order to boost adoption of PHRs and their role in integrated service delivery. Hastening of easy-to-adopt PHR-related standards and applications is needed to reduce administrative overhead and hesitance toward adoption for patient activation.
	Outreach	Increased efforts are needed to provide outreach and education that address the unique personal health management and communication needs of the underserved. Caregivers need to be equally educated so that they can become true ambassadors of health information technologies and their importance.
Policy	Funding and regulations	Health care reform of 2010 provides a major opportunity to extend PHR systems to underserved communities. Federal ARRA of 2009 and related policies need to advance significant PHR requirements and incentives that are inclusive of underserved populations. Federal ARRA of 2009 and related policies need to ensure that the privacy and confidentiality concerns of underserved communities are addressed.

Beginning with the personal domain noted in Table 1, it is identified that users will require that PHRs be multi-lingual (according to primary populations serviced), easy to navigate and use, and inherently respectful of privacy and security issues that might otherwise deter this population. To date, a Spanish speaking person would have a difficult time finding a PHR with Spanish translations that is supported in the US health care system even though the Spanish language is the second most common language in the United States.

Moving to the technical domain of the framework, there are also pervasive technical issues that underscore these personal constraints. Largest among these is a lack of widely adopted standards for the exchange of health data. Finding flexible, light-weight PHR systems that can be worked into a provider’s existing EMR system would be of invaluable assistance to having health information at the ready. Issues include provision of systems that can be effectively and inexpensively integrated with existing EMR systems while at the same time providing an interface that can clearly communicate health information to users. Furthermore, to reemphasize the aforementioned personal constraints, while a PHR technically provides the opportunity for extensive outreach abilities to patients, its effectiveness can be heavily undermined by not supporting the personal requirements (privacy, language, literacy, and access).

At the organizational domain level, the framework highlights how adoption of PHRs within public and private agencies, including community health organizations, may provide long-term financial benefits, but that in the short term it will necessitate extensive outreach and education efforts in order to influence not only the patients of these organizations, but their workers as well. It is possible that community health providers will have no choice but to adopt IT systems that can increase communications with their patients. A recent report by the California HealthCare Foundation noted that Medicare alone spends over US $12 billion per year for potentially preventable readmissions due to the inability to provide effective discharge and health education services [58]. Providers might be persuaded to adopt health information systems to help offset these enormous costs.

Finally, health care providers will only be able to adopt PHR systems if supported by policies that understand the unique issues encountered by community clinics and provide the funding needed to acquire the proper resources. This includes an understanding of how PHRs could be used to address public health issues and support inequities of Medicaid and Medicare systems. States are experiencing a continuing shortage of nurses for general care, decreases in the amount of doctors considering work in adult medicine and family practice, and declining provider participation in federal fee-for-service programs [59].

Exacerbating these issues are policies that have increased the number of restrictions on Medicaid reimbursement through federal laws and regulations. In relation to the PHR, if providers are unable to identify ways in which reimbursements can be obtained through PHR communications with patients, it is unlikely that their adoption will be seen as a feasible investment. Although there has been a national push toward adoption and integration of EMR systems as a whole, it is argued that the funding needed to allow community health organizations to do so in a manner that includes PHR capacity is yet to be adequate.

To sum up, this framework is meant to highlight considerations at the personal, technological, organizational, and policy levels that, if addressed, would facilitate the utilization of PHRs within underserved settings. While not a “recipe” for adoption, this framework does lay out general design guidelines for both technical design and broader organizational and policy design. Of course, achieving such adoption does not in and of itself translate into positive health outcomes. However, the emerging literature on PHRs does suggest that adoption has promise for health activation, communication, and improved health management [3,13,31,32]. For example, PHR pilot projects by organizations such as the Centers for Medicare and Medicaid Services [60] allow beneficiaries to choose from various PHRs (eg, Google Health, HealthTrio, NoMoreClipboard.com, and PassportMD) to assist in managing their care. The next wave of research would be to obtain a detailed understanding of the myriad health activity and health outcomes affected (or not) by active PHR use in underserved populations.

### Limitations

A limitation that should be noted is that although the patients and experts interviewed for this study provided a varied and broadly representative sample of the available expertise on the topic of underserved groups, this research did not presume to fully represent all of the possible subcategories of underserved communities that can be defined by language, culture, age, disability status, economic status, and other factors. It is clear that additional research is required to refine our understanding of how PHRs can address specific subgroups within these populations. The premise of the research also represented the most significant challenge, namely, that there has not been systematic adoption of PHRs in safety net settings. This meant that there was not significant existing literature or diverse implementations to draw upon. Moreover, many community clinics are currently struggling with basic health IT systems and the notion of a PHR can seem as an “out year” consideration. This context represented both a challenge and an opportunity for the research.

### Conclusion: Toward Positive Health Outcomes for All

ARRA of 2009 contained a major push for health IT, and this has been followed up by broader and historic health care reform. While this legislation has provided an unprecedented level of support for health IT, the act does not specifically target solutions for underserved populations. Yet, as discussed throughout this paper, there is a promising business, policy, and social case for using electronic services to enhance patient self-management among underserved populations. There are significant resources within ARRA of 2009 that can provide an important opportunity to extend electronic personal health records and services to underserved communities, such as through community health centers.

While the resources within ARRA of 2009 provide a general context for change, in and of themselves they are not sufficient to achieve the utilization that is warranted in underserved communities. As suggested by the framework, there are top-down, bottom-up, and midlevel needs that should be attended to in order to facilitate utilization. Self-empowerment through personal health technologies provides the opportunity to improve not only the health and welfare of the patient but also the fiscal and social health of society. Underserved groups are subject to what has been described as an “inverse information law” that limits access to information for those who need it most [61]. The multi-leveled set of actions outlined provides a means to create a level playing field for patient self-management through PHRs. The current wave of health IT support provides an important opportunity for leadership in designing and implementing PHR systems that can attend to the needs of all citizens in the United States and, in so doing, may offer insights for international efforts as well.

## Abbreviations

AHRQ: Agency for Health care Research and Quality
AARA: American Reinvestment and Recovery Act
ATM: automated teller machine
EMR: electronic medical record
HIPAA: Health Insurance Portability and Accountability Act
health IT: health information technology
MUA: medically underserved area
PHR: personal health record

## References

[1] Moiduddin A, Moore J (2008). US Department of Health and Human Services.

[2] (2009). World Health Organization.

[3] Kanaan SB (2009). California HealthCare Foundation.

[4] Weinick R, Shin P (2004). Agency for Healthcare Research and Quality.

[5] Agency for Healthcare Research and Quality (2009). US Department of Health and Human Services.

[6] Tang Paul C, Lansky David (2005). The missing link: bridging the patient-provider health information gap. Health Aff (Millwood).

[7] Marchioni G, Rimer B, Wildemuth B (2007). University of North Carolina.

[8] (2004). Markle Foundation.

[9] Tang Paul C, Ash Joan S, Bates David W, Overhage J Marc, Sands Daniel Z (2006). Personal health records: definitions, benefits, and strategies for overcoming barriers to adoption. J Am Med Inform Assoc.

[10] Jimison Holly, Gorman Paul, Woods Susan, Nygren Peggy, Walker Miranda, Norris Susan, Hersh William (2008). Barriers and drivers of health information technology use for the elderly, chronically ill, and underserved. Evid Rep Technol Assess (Full Rep).

[11] Halvorsson G (2007). Health Care Reform Now! A Prescription for Change.

[12] Brennan P F, Strombom I (1998). Improving health care by understanding patient preferences: the role of computer technology. J Am Med Inform Assoc.

[13] Weitzman Elissa R, Kaci Liljana, Mandl Kenneth D (2009). Acceptability of a personally controlled health record in a community-based setting: implications for policy and design. J Med Internet Res.

[14] Garrido Terhilda, Jamieson Laura, Zhou Yvonne, Wiesenthal Andrew, Liang Louise (2005). Effect of electronic health records in ambulatory care: retrospective, serial, cross sectional study. BMJ.

[15] Lafky D, Tulu B, Horan T (2006). Information systems and health care X: A user-driven approach to personal health records. Communications of the Association for Information Systems.

[16] Rosenbaum S, Jones E, Shin P, Ku L (2009). George Washington University School of Public Health and Health Services.

[17] (2007). California Budget Project.

[18] DeNavas-Walt C, Proctor B, Smith J (2008). US Census Bureau.

[19] Milicevic I, Cullen I (2005). IPSI.

[20] Andreassen Hege K, Bujnowska-Fedak Maria M, Chronaki Catherine E, Dumitru Roxana C, Pudule Iveta, Santana Silvina, Voss Henning, Wynn Rolf (2007). European citizens' use of E-health services: a study of seven countries. BMC Public Health.

[21] Kummervold Per Egil, Chronaki Catherine E, Lausen Berthold, Prokosch Hans-Ulrich, Rasmussen Janne, Santana Silvina, Staniszewski Andrzej, Wangberg Silje Camilla (2008). eHealth trends in Europe 2005-2007: a population-based survey. J Med Internet Res.

[22] Milicevic I, Gareis K, Korte WB Making progress towards user-orientation in online public service provision in Europe. http://www.eusereu.org/SHOWUSERSQL.asp?SQLID=3,2,4&show=LIST&MenuID=108.

[23] (2007). California HealthCare Foundation.

[24] Wallace Steven P, Gutiérrez Verónica F, Castañeda Xóchitl (2008). Access to preventive services for adults of Mexican origin. J Immigr Minor Health.

[25] Chang Betty L, Bakken Suzanne, Brown S Scott, Houston Thomas K, Kreps Gary L, Kukafka Rita, Safran Charles, Stavri P Zoe (2004). Bridging the digital divide: reaching vulnerable populations. J Am Med Inform Assoc.

[26] Harkin T (2009). Tom Harkin Iowa’s Senator.

[27] Botts N, Horan TA (2008). Bridging care communication and health management within diverse and underserved populations. http://aisel.aisnet.org/amcis2008/15.

[28] Strauss A, Corbin J (1998). Basics of Qualitative Research:Techniques and Procedures for Developing Grounded Theory. Second edition.

[29] Winkelman W (2005). Patient-perceived usefulness of online electronic medical records: Employing grounded theory in the development of information and communication technologies for use by patients living with chronic illness. J Am Med Inform Assoc.

[30] Botts N, Horan TA (2007). Electronic personal health records and systems to improve care for vulnerable populations. http://aisel.aisnet.org/amcis2007/116.

[31] Botts N, Thoms B, Horan T HealthATM: Interactive services for underserved populations. http://symposium2009.amia.org/files/symposium2009/AMIA_Advanced_Program_Final_LoRes.pdf.

[32] Moreno L, Peterson S, Bagchi A, Ursin R (2007). Mathematica Policy Research, Inc.

[33] Berland G K, Elliott M N, Morales L S, Algazy J I, Kravitz R L, Broder M S, Kanouse D E, Muñoz J A, Puyol J A, Lara M, Watkins K E, Yang H, McGlynn E A (2001). Health information on the Internet: accessibility, quality, and readability in English and Spanish. JAMA.

[34] Molnar C (2000). Quality Interagency Coordination Task Force.

[35] Atkinson Nancy L, Saperstein Sandra L, Desmond Sharon M, Gold Robert S, Billing Amy S, Tian Jing (2009). Rural eHealth nutrition education for limited-income families: an iterative and user-centered design approach. J Med Internet Res.

[36] Hesse Bradford W, Shneiderman Ben (2007). eHealth research from the user's perspective. Am J Prev Med.

[37] Shneiderman B (2000). Universal usability. Communications of the ACM.

[38] Clancy Carolyn M, Kiley James P, Weiss Kevin B (2007). Eliminating asthma disparities through multi-stakeholder partnerships. Chest.

[39] (2008). Deloitte Center for Health Solutions.

[40] Goslee S, Conte C (1998). Benton Foundation.

[41] Rohde GL, Shapiro R (2000). US Department of Commerce.

[42] Brown J (2003). Crossing the Digital Divide. Ermann MD, Shauf MS, editors. Computers, Ethics and Society. 3rd edition.

[43] Lievrouw L, Farb S (2003). Information and Equity. Cronin B, editor. Annual Review of Information Science and Technology.

[44] Berkman N D, Dewalt D A, Pignone M P, Sheridan S L, Lohr K N, Lux L, Sutton S F, Swinson T, Bonito A J (2004). Literacy and health outcomes. Evid Rep Technol Assess (Summ).

[45] Brodie M, Flournoy R E, Altman D E, Blendon R J, Benson J M, Rosenbaum M D (2000). Health information, the Internet, and the digital divide. Health Aff (Millwood).

[46] Martin Z (2006). Underserved clinics collaborate on I.T. Health Data Manag.

[47] Rogers E (2003). Diffusion of Innovations. 5th edition.

[48] Valleta R, MacDonald G (2003). Federal Reserve Bank of San Francisco Economic Letter.

[49] Kim Eung-Hun, Stolyar Anna, Lober William B, Herbaugh Anne L, Shinstrom Sally E, Zierler Brenda K, Soh Cheong B, Kim Yongmin (2009). Challenges to using an electronic personal health record by a low-income elderly population. J Med Internet Res.

[50] Compaine B (2001). Stakes for Democracy. Compaine BM, editor.

[51] Salvador T, Sherry J (2004). Local Learnings: An essay on designing to facilitate effective use of ICTs. The Journal of Community Informatics.

[52] (2008). California HealthCare Foundation.

[53] Botts N, Thoms B, Noamani A, Horan T (2010). Cloud computing architectures for the underserved: Public health cyberinfrastructures through a network of HealthATMs. http://doi.ieeecomputersociety.org/10.1109/HICSS.2010.107.

[54] 111th Congress (2009-2010) GovTrack.us.

[55] (2010). Centers for Medicare & Medicaid Services.

[56] Arzt N (2007). State of Utah Department of Health.

[57] Wiljer David, Urowitz Sara, Apatu Emma, DeLenardo Claudette, Eysenbach Gunther, Harth Tamara, Pai Howard, Leonard Kevin J, Canadian Committee for Patient Accessible Health Records (2008). Patient accessible electronic health records: exploring recommendations for successful implementation strategies. J Med Internet Res.

[58] (2008). California HealthCare Foundation.

[59] (2008). California Department of Health Care Services.

[60] (2005). Centers for Medicare & Medicaid Services.

[61] Eysenbach Gunther (2007). Poverty, human development, and the role of eHealth. J Med Internet Res.

